# Omental Infarction Imitating Acute Appendicitis

**DOI:** 10.7759/cureus.8704

**Published:** 2020-06-19

**Authors:** Saurabh Gaba, Nayana Gaba, Monica Gupta

**Affiliations:** 1 General Medicine, Government Medical College and Hospital, Chandigarh, IND; 2 Obstetrics and Gynaecology, Postgraduate Institute of Medical Education and Research, Chandigarh, IND

**Keywords:** omental infarction, acute appendicitis, acute abdomen

## Abstract

A 26-year-old male patient with no significant past history presented with a two-day illness of nausea and abdominal pain, mimicking acute appendicitis. The appendix was poorly visualized on the ultrasound scan so a CT scan was done which revealed infarction of the omentum on the right side of the abdomen. The patient was closely monitored and managed conservatively with analgesics, fluids and antibiotics. Spontaneous improvement occurred in a day, and oral feeding was resumed. The clinical course was uncomplicated, and the patient was discharged, circumventing unnecessary surgery. Literature search has revealed that omental infarction is a rare cause of acute abdomen and it can mimic acute appendicitis or cholecystitis. The treatment needs to be individualized, and surgery may or may not be required.

## Introduction

Acute appendicitis is one of the most common causes of an acute abdomen presenting to the emergency departments worldwide [1]. In most of the cases, diagnosis can be made easily due to the stereotypical clinical features and radiological findings. The patient presents with visceral umbilical pain which shifts to the right lower abdomen once the parietal peritoneum in involved. It is accompanied with anorexia, nausea, vomiting and characteristic tenderness at the McBurney’s point [1]. However, there are varied etiologies of presentation similar to acute appendicitis which may make the diagnosis obscure and challenging. We are reporting a case of idiopathic omental infarction which created diagnostic confusion as the presentation was similar to acute appendicitis. Timely diagnosis made on CT scan helped avoid a needless surgery.

## Case presentation

A 26-year-old male patient developed non-colicky pain in the right lower abdomen which reached its zenith in two days. It was associated with nausea and mild fever, but he had no trouble passing stools and flatus. There was no history of any dysuria or dysentry. He walked into the emergency room unaided. Examination revealed a lean individual with a pulse of 100 beats per minute, a blood pressure of 130/70 mmHg and an axillary temperature of 100°F. Abdomen was soft, albeit with tenderness in the right lumbar and iliac region. Bowel sounds were normal and per rectal examination was unremarkable. Bilateral testes were normal, and there were no signs of epididymo-orchitis. There was no significant past medical or surgical history. At this point, the clinical differential diagnoses considered were acute appendicitis, acute cholecystitis, acute pyelonephritis, ureterolithiasis, bacterial enteritis or typhlitis and amebic colitis.

Initial blood investigations displayed an acute phase response with leukocytosis (14 × 10^9^/L) and elevated C-reactive protein (39 mg/dL). The hemoglobin and platelet indices were within normal range. The renal and hepatic functions were normal, and serum amylase and lipase were not elevated. Urine analysis and microscopy were normal. Stool examination did not reveal any ova or cyst. Ultrasound of the abdomen reported the finding of heterogeneously hyperechoic mesentery in the right iliac fossa and lumbar region with mild free fluid, and non-visualization of appendix separately. These findings were inconclusive and did not provide a definite diagnosis. Management till this point included intravenous fluids, parenteral ciprofloxacin (200 mg BID), metronidazole (500 mg TID) and tramadol (100 mg BID). The surgical team was of the view that a CT scan be done before undertaking any surgical intervention.

**Figure 1:**
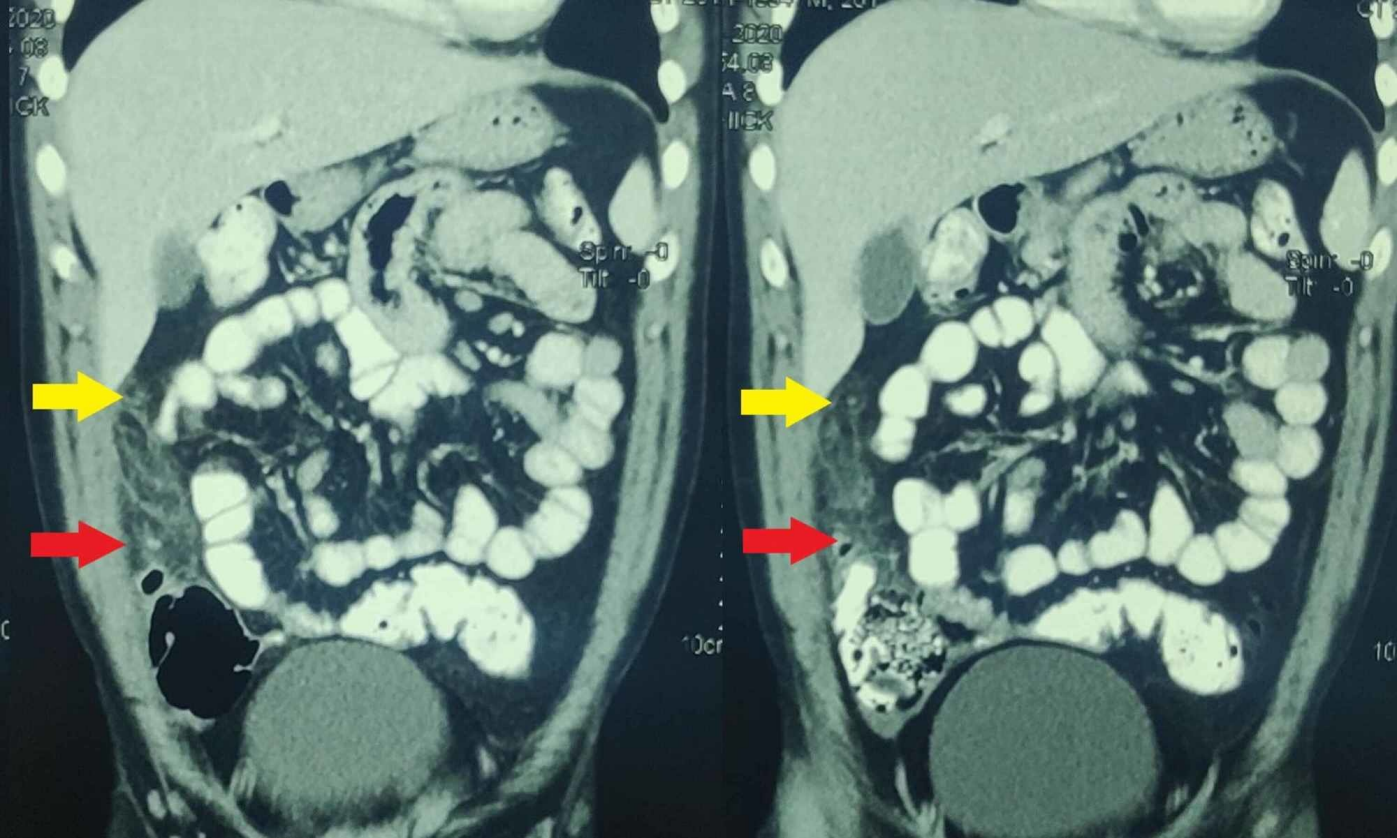
Coronal sections of contrast-enhanced CT scan of the abdomen. There is an elongated oval-shaped mixed fat and soft tissue density (red arrows) measuring 4.4 × 2.1 × 6.0 cm in the right side of abdomen, signifying omental infarction. The lesion is extending from right iliac fossa to the subhepatic region (yellow arrows).

**Figure 2:**
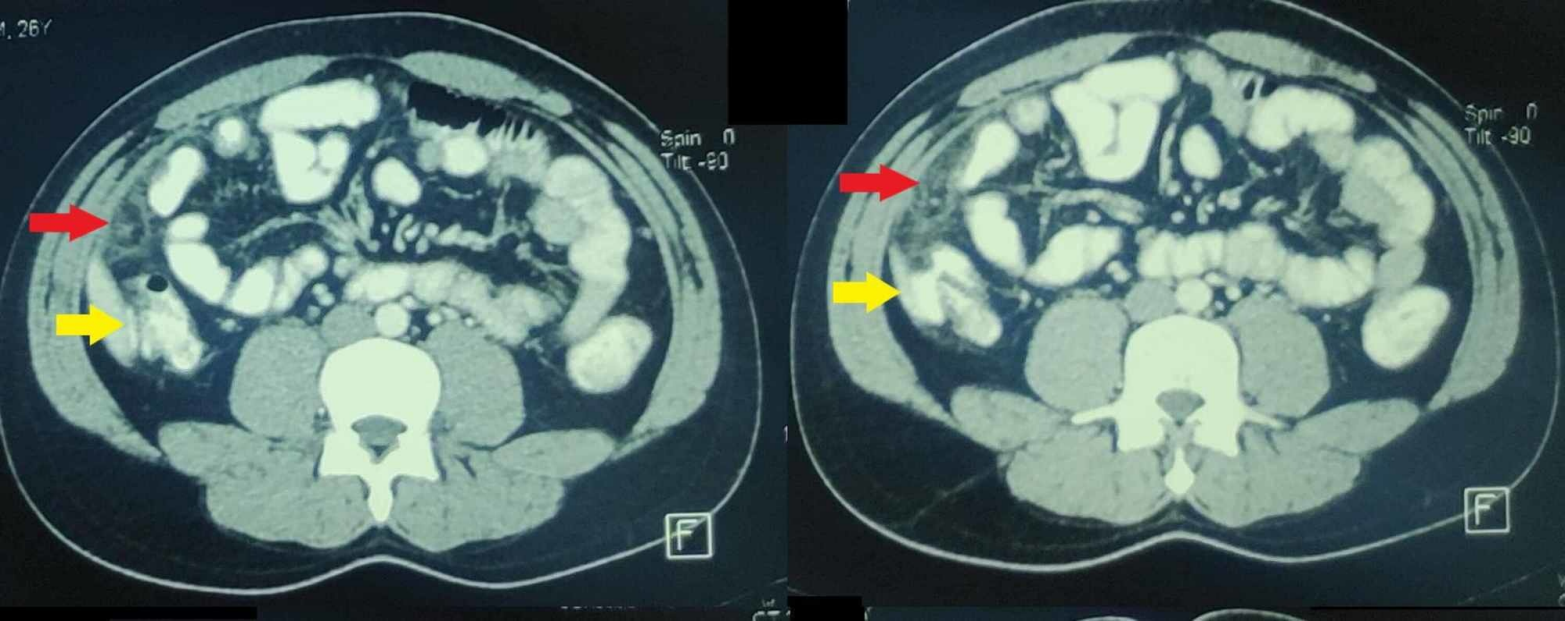
Axial sections of contrast-enhanced CT scan of the abdomen. The lesion produced by omental infarction (red arrows) can be seen anterior to the ascending colon (yellow arrows).

An emergent contrast-enhanced CT scan (Figures 1, 2) was done.

Omental infarction was diagnosed, and normal appendix was visualized separately. Conservative management was continued, and the patient’s vitals and inflammatory markers were closely monitored. Clinical improvement was noted within 12 hours, and oral feeding was resumed. He was discharged after three days with a prescription of analgesics and antibiotics. On follow-up appointment a week later, he was found to be completely asymptomatic with no abnormality on the abdominal examination; hence, a repeat CT was not performed.

The infarction could not be attributed to any etiology. There was no history of abdominal trauma. A thorough search was carried out to look for any vasculitic and thrombophilic disorders. Antinuclear antibodies (ANA), antineutrophil cytoplasmic antibodies (ANCA) and antiphospholipid antibodies (APLA) were not detected. Prothrombin and activated partial thromboplastin times were normal. Levels of protein C, protein S and antithrombin III were within normal range, and factor V mutation was not identified.

## Discussion

Omentum is a fat laden structure lying anteriorly in the abdominal wall [2]. The greater omentum hangs from the greater curvature of the stomach and the lesser omentum extends from the liver to the lesser curvature of the stomach. Anatomically, it is a fold of the visceral omentum, and serves immunological function by providing a physical barrier for the internal organs and by housing macrophages [2].

Infarction of the omentum is an uncommon phenomenon and has been known to mimic other surgical conditions, such as acute appendicitis and cholecystitis [3]. Itenberg et al. have reported that close to 0.4% of cases treated as appendicitis actually have omental infarction [4]. Obesity is known to be risk factor [5]. This may be related to compression of blood vessels by the surrounding fat. Other associations are trauma and intense exercise [6]. For unknown reasons, omentum on the right side of the abdomen has been found to be more prone to infarction, probably the precarious arterial supply of the right edge of the greater omentum may explain this phenomenon [7].

Lindley and Peyser encountered a case similar to the one described in this report [8]. The CT finding was of inflammatory stranding adjacent to cecum and ascending colon. In view of persistent fever, they proceeded to diagnostic laparoscopy and visualized the necrotic omentum. No intrusion was done since it was perceived that collateral damage to neighboring structures during removal of devitalized omentum would increase the morbidity. Expectant management resulted in complete recovery, and the patient was discharged three days later. Park et al. have described a case series of four patients with omental infarction [9]. Three of them responded to conservative treatment and one required partial omentectomy after developing systemic dysfunction. In the case of a seven-year-old boy described by Siddiqui et al., the CT scan displayed inflammatory changes in the right iliac fossa which incorporated the appendix along with omentum. This necessitated a laparotomy which confirmed omental infarction [10]. Balthazar et al. have reported a case of left-sided omental torsion leading to infarction and abscess formation that needed open surgical debridement [11]. Infarction of the whole omentum has also been documented in the 27-year-old male patient secondary to torsion around right inguinal hernia [12]. He was successfully managed conservatively.

## Conclusions

Omental infarction is a rare but plausible cause of right-sided abdominal pain, and awareness about this condition can be valuable for surgeons as well as physicians. Diagnosis is not possible on clinical grounds alone as the condition is a great impersonator. Emergency abdominal ultrasonography might be suboptimal; hence, a contrast-enhanced CT is preferred for confirmation of the diagnosis. No definite guidelines exist for management, but anecdotal data suggest that a trial of non-surgical management is reasonable for most patients, provided they are closely monitored.
